# Learning touch preferences with a tactile robot using dopamine modulated STDP in a model of insular cortex

**DOI:** 10.3389/fnbot.2015.00006

**Published:** 2015-07-22

**Authors:** Ting-Shuo Chou, Liam D. Bucci, Jeffrey L. Krichmar

**Affiliations:** ^1^Department of Computer Sciences, University of California, IrvineIrvine, CA, USA; ^2^Department of Cognitive Sciences, University of California, IrvineIrvine, CA, USA

**Keywords:** tactile robot, reinforcement learning, dopamine, STDP, insular cortex, somatosensory cortex

## Abstract

Neurorobots enable researchers to study how behaviors are produced by neural mechanisms in an uncertain, noisy, real-world environment. To investigate how the somatosensory system processes noisy, real-world touch inputs, we introduce a neurorobot called CARL-SJR, which has a full-body tactile sensory area. The design of CARL-SJR is such that it encourages people to communicate with it through gentle touch. CARL-SJR provides feedback to users by displaying bright colors on its surface. In the present study, we show that CARL-SJR is capable of learning associations between conditioned stimuli (CS; a color pattern on its surface) and unconditioned stimuli (US; a preferred touch pattern) by applying a spiking neural network (SNN) with neurobiologically inspired plasticity. Specifically, we modeled the primary somatosensory cortex, prefrontal cortex, striatum, and the insular cortex, which is important for hedonic touch, to process noisy data generated directly from CARL-SJR's tactile sensory area. To facilitate learning, we applied dopamine-modulated Spike Timing Dependent Plasticity (STDP) to our simulated prefrontal cortex, striatum, and insular cortex. To cope with noisy, varying inputs, the SNN was tuned to produce traveling waves of activity that carried spatiotemporal information. Despite the noisy tactile sensors, spike trains, and variations in subject hand swipes, the learning was quite robust. Further, insular cortex activities in the incremental pathway of dopaminergic reward system allowed us to control CARL-SJR's preference for touch direction without heavily pre-processed inputs. The emerged behaviors we found in this model match animal's behaviors wherein they prefer touch in particular areas and directions. Thus, the results in this paper could serve as an explanation on the underlying neural mechanisms for developing tactile preferences and hedonic touch.

## Introduction

Humans and other animals respond preferentially to different types of touches. For example most cats prefer to be petted from head to tail rather than the other way around. Although, tactile sensing is an active area of robotics research, which takes inspiration from biology and neuroscience, most tactile robots have been developed to sense the borders and shapes of objects (Pearson et al., 2011; Evans et al., 2012; Schroeder and Hartmann, 2012) or for grasping and detecting surfaces (Bologna et al., 2011, 2013; Spigler et al., 2012). The present paper introduces a tactile neurorobot that has a surface designed for petting. The robot has the ability to signal its preferences through coloration of its surface and auditory signals. We will use this neurorobot to explore neural mechanisms of learning in uncertain, real-world environments.

In a set of mutual reinforcement learning experiments, we demonstrate that a user can pair colors on the robot's surface with hand sweeps in preferred directions across the robot's surface. The spatiotemporal nature of tactile stimuli, as well as the noisy sensors and environments, in which they operate, make the perception of touch a complex problem. To address these issues, we introduce a biologically spiking neural network, which learns through a novel dopaminergic spike timing dependent plasticity mechanism. Specifically, the robot has built-in tactile preferences and a user must learn these preferences, as well as reward the robot by touching the robot in its preferred ways. Auditory tones were used to signal pleasure and disappointment. In this way, the robot can learn the association between a color and a gentle touch.

Because the neurorobot's main sensory modality was touch, we developed a neurobiologically plausible model of tactile sensing. Mammals have two tactile pathways; one which is fast and delivers fine touch resolution, and another which is slower with coarser resolution that delivers hedonic or value-laden touch information. It is this latter touch pathway that we will explore in the present experiments. In animals, sophisticated cutaneous mechanoreceptors in the skin are capable of perceiving temperature, indentation, stretch, vibration, and movement (Abraira and Ginty, 2013). Unlike primary visual cortex for visual information, primary somatosensory cortex (S1) is not the only first order cortical region processing tactile information from thalamus (TH). Insular cortex (IC), which was thought to be higher hierarchical cortical region receiving tactile information from secondary somatosensory cortex (S2) (Felleman and Van Essen, 1991), also processes tactile information ascending directly from posterior ventromedial thalamus (VMpo) in macaque (Sewards and Sewards, 2002) and human (Craig et al., 1994). The parallel pathways to insular cortex in mammals imply that a single piece of tactile information could be heterogeneously processed in different regions and then integrated in insular cortex. For instance, a gentle touch detected by mechanoreceptors with C-fiber and Aβ-fiber triggers spike trains going through TH→IC and TH→S1→S2→IC pathways, respectively. The spike trains invoke pleasant sensation, which is correlated with insular activity (Morrison et al., 2011a,b). The pleasant sensation could be a state of emotional representation in anterior insular cortex (AIC) as a result of integrating tactile information along posterior insular (pIC), mid-insular (mIC) and anterior insular (AIC) (Craig, 2002, 2009). In the present study, we are interested in the neural mechanism for integrating tactile information in this area because the unconscious element of the pleasant sensation might link to the dopamine system (Schultz, 2006) and therefore defines innate preferences or values (Krichmar and Rohrbein, 2013).

To explore learning mechanisms for hedonic touch, we constructed a spiking neural network (SNN) model of the posterior insular cortex (pIC), somatosensory cortex, and the areas necessary for value-based learning. The neural dynamics in the model of pIC accounted for: (1) the robot's tactile preferences, (2) processing real-world tactile inputs with minimal pre-processing, (3) demonstrating that wave propagation is a viable means to generating precise spike timing in the face of noise, and (4) learning associations between neutral stimuli and hedonic touch.

Because hedonic touch requires a caresser and a caressee, we developed a human robot interaction study that required mutual reinforcement learning. To achieve these goals, we built a robot, named CARL-SJR (Cognitive Anteater Robotics Laboratory—Spike Judgment Robot), with a large tactile sensory area and a surface capable of displaying bright colors. CARL-SJR's behavior was controlled by the dual-pathway model (Brown et al., 1999; Tan and Bullock, 2008; Chorley and Seth, 2011), which minimizes prediction error signaled by dopamine (Schultz, 2006).

## Materials and methods

### CARL-SJR neurorobot

**Figure 1:**
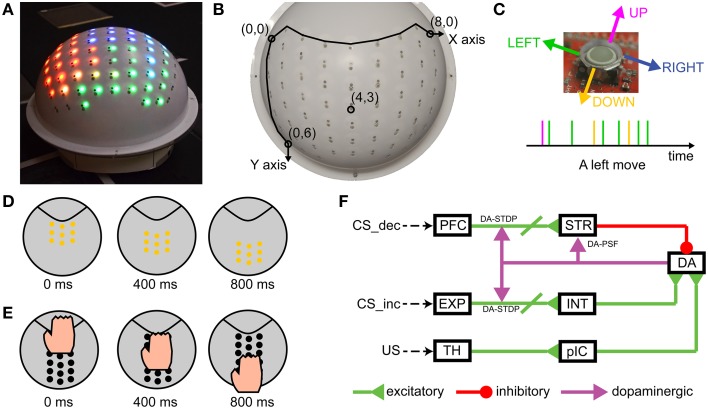
**CARL-SJR is an interactive, tactile neurorobot. (A)** Photograph of CARL-SJR. The shell has a 9-by-8 matrix of trackballs each collocated with red, green, and blue color LEDs. Any animated color pattern is possible. **(B)** The 9-by-8 matrix of trackballs forms the coordinate system of CARL-SJR. The most up left trackball is mapped to the origin (0, 0) while the most down right trackball is mapped to the coordinates (8, 6). **(C)** A trackball can detect a touch event in four directions. Assuming a trackball rolls in the left direction, a sequence of touch events will be generated as shown by the timeline. The majority of touch events will be left events with events in other directions due to sensor noise. **(D)** An example of an animated color pattern. The yellow pattern moves downward in 800 ms. **(E)** An example of a typical touch pattern. The hand moves downward. Usually 4 ~ 7 trackballs are touched simultaneously. **(F)** Schematic of the spiking neural network architecture that controlled CARL-SJR's behavior.

The sensory encoding and learning experiments were conducted with a novel robot named CARL-SJR. CARL-SJR is autonomous, mobile, self-contained, and capable of tactile sensing and interaction (see Figure 1). To give the robot a sense of touch, we incorporated an array of trackballs, which are typically found in cellphones and other devices. The trackball array can signal the direction and velocity of tactile stimuli. The robot's unique form factor encourages users to rub or pet its surface. The robot has LEDs co-located at each trackball, which can display a wide range of colors in response to touch. CARL-SJR has a large tactile sensory area and the ability to display bright colors on its shell (see Figure 1A). CARL-SJR's shell has a 9-by-8 matrix of true color LEDs. Any animated color pattern can be programmed to display on its shell. The shell also has a 9-by-8 matrix of trackballs, which are used for sensing tactile input. The trackballs form a coordinate system with the upper left trackball mapped to the origin (0, 0), and the downward right trackball mapped to the maximum coordinates (8, 6) (see Figure 1B). A trackball can detect a touch event in four directions (i.e., up, down, left, and right). Assuming a trackball rolls in the left direction, a sequence of touch events will be generated as shown by the timeline in Figure 1C. In this example, the majority of touch events are in the left direction accompanied by noise in other directions. The current version of CARL-SJR mounted the tactile shell on an iRobot Create platform. A computer, which communicated with CARL-SJR over Bluetooth, executed the neural model, collected trackball data, controlled the LEDs on the shell, and controlled the motors and speakers on the iRobot Create. More details on the robot hardware can be found at Bucci et al. (2014).

CARL-SJR displayed color patterns as output, and took hand movements as input. The display patterns could be a solid color, mixed, or animated. Figure 1D shows an example of an animated color pattern. The yellow pattern moves downward in 800 ms. Hand movements across the shell triggered touch events in the matrix of trackballs. Figure 1E shows a typical touch pattern. The hand moves downward. Usually 4 ~ 7 trackballs are touched simultaneously.

### Spiking neural network model

To support the present mutual reinforcement learning experiments, we built a spiking neural network (SNN) model using the large scale SNN simulator CARLsim to recognize tactile sensory input, and to control CARL-SJR's behavior (Nageswaran et al., 2009; Richert et al., 2011; Carlson et al., 2014). CARLsim was written in C/C++/CUDA and designed to leverage the parallel computing power of GPUs. The present SNN model had 13,000 neurons and 200,000 synapses and could run four times faster than real time. However, the model was slowed down to match the real time robotic application.

The SNN was designed to be biologically plausible and simulated somatosensory pathways, as well as neurally inspired learning. To support learning, we implemented a variation of the dual-pathway model (Brown et al., 1999; Tan and Bullock, 2008; Chorley and Seth, 2011), which minimizes prediction error signaled by dopamine (Schultz, 2006). In the present experiments, the Conditioned Stimulus (CS) was a color pattern displayed on CARL-SJR's surface, and the Unconditioned Stimulus (US) was a touch pattern initiated by the user sweeping his or her hand across CARL-SJR's surface. The decremental (dopamine) pathway for the CS (see PFC→STR→DA in Figure 1F) decreased DA neurons' activity through inhibitory projections. In contrast, the incremental (dopamine) pathway for the US (see TH→pIC→DA in Figure 1F) increased spikes of dopaminergic neurons. The complementary pathways converge on a group of DA neurons and control the DA response. Phasic neural activity in the incremental pathway for US might change the balance of excitation and inhibition, thus triggering a DA burst, which in turn signals striatum (STR) and PFC→STR synapses through dopaminergic projections. The decremental pathway is able to learn the timing of US and then increases inhibitory force on DA neurons for restoring the balance. The neural activities in prefrontal cortex (PFC) and striatum are crucial for learning the timing of US. Chorley and Seth's model incorporated pre-generated polychronous groups (Izhikevich, 2006) for precisely timed spikes in PFC. However, polychronous groups are a theoretical prediction and, to the best of our knowledge, have not been shown empirically. Moreover, it would be difficult to show repeatability of this precise timing in a computational model having noisy and uncertain inputs. Rather than relying on precisely timed polychrony or synfire chains, we used wavelike neural activity for propagating information through the simulated brain regions. These waves of neural activity have empirical support and do not require precisely timed spike sequences (Rubino et al., 2006; Benucci et al., 2007; Ferezou et al., 2007; Han et al., 2008; Wu et al., 2008; Lubenov and Siapas, 2009; Sato et al., 2012).

#### Spiking neuron model

CARLsim incorporated a phenomenological model of a spiking neuron proposed by Izhikevich (2003). The dynamics of each neuron is governed by the following equations:

(1) $$\dot{v}=0.04v^{2}+5v+140-u+I$$

(2) $$\dot{u}=a\left(bv-u\right)$$

The variable *v* is the membrane potential of a neuron and the variable *u* is an abstract membrane recovery current. The variable *I* is the input current (i.e., the current flow into a neuron). A neuron emits a spike if its membrane potential is higher than 30 mv and then resets according to the following equation:

(3) $$if\;v\geq 30,\;then\;\{\begin{array}{c} v=c\\ u=u+d \end{array}$$

Both excitatory regular spiking (RS) neurons and inhibitory fast spiking (FS) neurons were used in the model. For RS neurons, we set *a* = 0.02, *b* = 0.2, *c* = −65.0, and *d* = 8.0. For FS neurons, we set *a* = 0.1, *b* = 0.2, *c* = −65.0, and *d* = 2.0. For more biologically realistic dynamics, a conductance synapse model was used to calculate the input current for each neuron (Izhikevich and Edelman, 2008). The equation is:

(4) $$\begin{array}{l} I=g_{AMPA}\left(0-v\right)+g_{NMDA}\frac{{\left[\frac{-80-v}{60}\right]}^{2}}{1+{\left[\frac{-80-v}{60}\right]}^{2}}\left(0-v\right)\\ +g_{GABA_{A}}\left(-70-v\right)+g_{GABA_{B}}(-90-v) \end{array}$$

where *v* is again the membrane potential and *g* is the total conductance for ion channels created by different receptors (i.e., AMPA, NMDA, GABA_A_, GABA_B_). The conductance *g* is increased by the amount of synaptic weight *w* upon the arrival of a spike and decays along time as described by the equations below:

(5) $$g_{i,\;k}\;=\sum_{j}^{N}w_{jk}\delta (t-t_{pre,\;j}),\;i\in \{AMPA,\;NMDA,\;GABA_{A},\;GABA_{B}\}$$

(6) $${\dot{g}}_{i,\;k}=-\frac{g_{i,\;k}}{\tau_{i}}\;i\in \{AMPA,\;NMDA,\;GABA_{A},\;GABA_{B}\}$$

where *N* is the number of pre-synaptic neurons and *w*_*jk*_ is the weight of the synapse connecting pre-synaptic neuron *j* and post-synaptic neuron *k*. δ is the Dirac delta function. *t* is the current time (i.e., current simulation time step when we approximate the continuous function in discrete time steps) and *t*_*pre, j*_ is the arrival time of the last spike from neuron *j*. The decay constant τ_*i*_ was set to 5, 100, 6, and 150 ms for different receptors AMPA, NMDA, GABA_A_, and GABA_B_, respectively.

#### STDP, DA-STDP and DA-PSF

Spike timing dependent plasticity (STDP) (Caporale and Dan, 2008; Markram et al., 2011) was applied to excitatory and inhibitory synapses in the computational model. In our model, excitatory STDP and inhibitory STDP were used to develop and stabilize wavelike neural activity in the PFC area (see Section Wave Propagation in PFC). The synaptic weights were governed by the following equations:

(7) $${\dot{w}}_{exc}=A^{+}e^{\frac{t_{pre}-t_{post}}{\tau_{+}}}\delta (t-t_{post})-A^{-}e^{\frac{t_{post}-t_{pre}}{\tau_{-}}}\delta (t-t_{pre})$$

(8) $$\begin{array}{l} {\dot{w}}_{inh}=B^{+}H^{+}\left(\left|t_{post}-t_{pre}\right|\right)\delta \left(t-t_{pre}\right)\\ +B^{+}H^{+}\left(\left|t_{post}-t_{pre}\right|\right)\delta \left(t-t_{post}\right)\\ -B^{-}H^{-}\left(\left|t_{post}-t_{pre}\right|\right)\delta \left(t-t_{pre}\right)\\ -B^{-}H^{-}\left(\left|t_{post}-t_{pre}\right|\right)\delta \left(t-t_{post}\right) \end{array}$$

(9) $$H^{+}\left(x\right)=\{\begin{array}{c} 1,\;if\;0<x\leq \lambda \\ 0,\;otherwise \end{array}\text{​​​},\;H^{-}\left(x\right)=\{\begin{array}{c} 1,\;if\;\lambda <x\leq \;\gamma \\ 0,\;otherwise \end{array}$$

The variable *w*_*exc*_ is an excitatory synaptic weight. *t*_*pre*_ is the arrival time of last pre-synaptic spikes while *t*_*post*_ is the time of post-synaptic spikes. δ is again the Dirac delta function. We set the E-STDP parameters *A*^+^*/A*^−^ and τ_+_/τ_−_ to 0.1/0.07 and 20/40 ms respectively. The variable *w*_*inh*_ is an inhibitory synaptic weight. The I-STDP were modeled as a piecewise linear Mexican hat function as was described in Srinivasa and Jiang (2013). The value of |*t*_*pre*_*-t*_*post*_|determines LTP or LTD. λ and γ define the ranges of inhibitory LTP and LTD. We set the I-STDP parameters *B*^+^*/B*^−^ and λ/γ to 0.1/0.06 and 4/20 ms.

Dopamine modulated spike timing dependent plasticity (DA-STDP) served as the underlying neural mechanism for reinforcement learning and solving the distal reward problem (Izhikevich, 2007). Both the incremental and decremental pathways for CS receive dopamine signals (see Figure 1F) and their synapses are subject to DA-STDP. In this form, the E-STDP function does not directly change synaptic weights, but instead modulates weights through an eligibility trace. The change of eligibility trace *c*, dopamine value *d*, and excitatory synaptic weight *w*_*exc*_ are described by the following equations:

(10) $$\begin{array}{l} \dot{c}=-c/\tau_{c}+A^{+}e^{\frac{t_{pre}-t_{post}}{\tau_{+}}}\delta (t-t_{post})\\ -A^{-}e^{\frac{t_{post}-t_{pre}}{\tau_{-}}}\delta (t-t_{pre}) \end{array}$$

(11) $$\dot{d}=-d/\tau_{d}+da_{syn}\delta (t-t_{pre})$$

(12) $${\dot{w}}_{exc}=cd$$

The excitatory synaptic weight *w*_*exc*_ is scaled by variable *d*, which is the dopamine concentration of the target neural group (i.e., the post neural group projected by dopaminergic synapses). The dopamine value *d* is increased by *da*_*syn*_, which is 0.04, for each spike reaching the target neural group. The value of *d* ranges from the baseline value 1.0 μM to a peak value 20.0 μM. Please note, both *d* and *c* decay over time. The symbol δ represents the Dirac delta function and is one if there is an action potential at *t*_*pre*_ and zero otherwise. The time constant τ_*c*_ and τ_*d*_ are 1000 and 50 ms as shown in Equations (10) and (11), respectively.

Dopamine modulated post-synaptic facilitation (DA-PSF) is the phenomenon where excitability of a post-synaptic neural group is modulated by dopamine (Nicola et al., 2000; Williams and Castner, 2006). A large portion of medium spiny neurons in striatum has D1 receptors to allow extra input current. We modeled this phenomenon as the following equation:

(13) $$g_{efc,\;i}=g_{i}\left(0.9+0.1d\right),\;i\in \{AMPA,\;NMDA\}$$

where *d* is the dopamine value and *g*_*efc*_ is the effective conductance used for calculating input current by Equation (4). Note that the minimum gain 1.0 is due to the baseline dopamine value of 1.0 μM.

#### Network architecture

**Figure 2:**
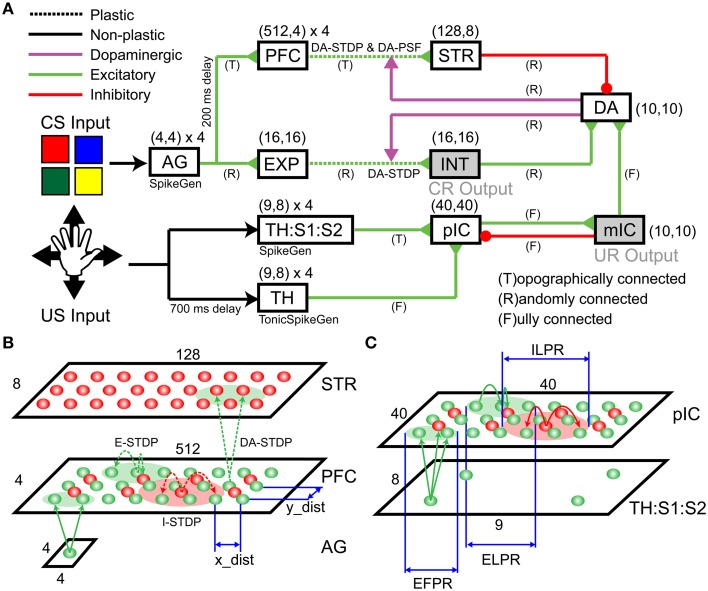
**Detailed Spiking Neural Network (SNN) architecture**. The SNN network model includes incremental (dopamine) pathway and decremental (dopamine) pathway. **(A)** An overview of network architecture. The model consists of an Action Generator (AG) group, a sensory experience (EXP) group, prefrontal cortex (PFC), striatum (STR), an intermediate area (INT), thalamus (TH), thalamus/somatosensory area (TH:S1:S2), posterior insular cortex (pIC), mid-insular cortex (mIC), and a group of dopaminergic neurons (DA). **(B)** Schematic of the projection patterns in the incremental pathway from AG to PFC to STR. **(C)** Schematic of the projection patterns in the decremental pathway from TH:S1:S2 to pIC.

The neural architecture for the present study, which is based on the dual-pathway model (Brown et al., 1999; Tan and Bullock, 2008; Chorley and Seth, 2011), is depicted in Figure 2A. Color information, from CARL-SJR's shell, which represents the CS, is fed into both the decremental and incremental pathways. The incremental pathway also signals tactile information, which represents the US. It has been suggested that the incremental and decremental pathways balance each other out so that after learning the dopamine signal is suppressed when a reward is predicted by the CS and arrives at the expected time with a US.

The decremental pathway for the CS goes from the ActionGenerator (AG), to the prefrontal cortex (PFC), to the striatum (STR), and then to a pool of dopaminergic neurons (DA). All connections in the decremental pathway are excitatory, except for the STR→DA connections, which are inhibitory. The cortical area AG is used to generate spike trains that encode color responses. AG has 64 excitatory neurons for four CS input channels (i.e., red, green, blue, and yellow). Each neuron in a channel, which has 16 neurons totally, emits a spike if the corresponding CS input is presented. Figure 2B illustrates more detailed connections of a channel from AG to PFC to STR. A pre-synaptic neuron in AG is topographically connected to a post-synaptic neuron in PFC based on the distance between neuron locations (see x_dist and y_dist in Figure 2B). The smaller green circle in Figure 2C defines the Excitatory Forward Projecting Radius (EFPR), which is the standard deviation of the Gaussian projection in spatial domain. Specifically, the distance between two neurons determines the connection probability (i.e., Gaussian distribution) and conductance delay (i.e., axonal delay). A channel in PFC has 2048 excitatory neurons and 512 inhibitory neurons. They are connected laterally according to Excitatory/Inhibitory Lateral Projecting Radius (ELPR/ILPR). The excitatory/inhibitory radius and synaptic weights were tuned to exhibit the behavior of traveling waves (Chen et al., 2013). If a CS input is presented, AG transmits spikes to the left side of the corresponding channel in PFC. The wide rectangular shape allows wavelike neural activity to propagate along the long side with precise timing. The idea behind the wave propagation in PFC is that the position of a neuron encodes the time relative to the release of a CS inputs (see Section Wave Propagation in PFC). PFC and STR are topologically connected according to EFPR as well. PFC→STR synaptic weights were subject to DA-STDP and sensitive to DA signals. The plastic PFC→STR synapses were able to learn (or predict) the timing of following US (see Section PFC-to-STR Synapses Learn to Predict the Timing of US). STR and DA are randomly connected and STR→DA synaptic weights were tuned to match the range of excitatory force from the incremental pathways.

The incremental pathway for the CS goes from AG to cortical area Expression (EXP), to Intermediate area (INT), and then to DA with excitatory projections. AG→EXP→INT→DA are randomly connected. EXP→INT connections are plastic synapses subject to DA-STDP and representations for CS inputs (i.e., red, green, blue, and yellow) will form in INT if a color pattern is reinforced (see Section CARL-SJR's Behaviors during Learning Multiple CS-US Pairs). INT→DA synaptic weights were tuned to match the range of inhibitory force from STR. INT was also linked to conditioned response (CR) in which CARL-SJR rotates its body if INT has more than 50 spikes within 50 ms.

For the incremental pathway, we implemented both the fast Aβ and the slower C-fiber tactile pathways. The incremental pathway for US starts with tactile inputs from touch events and projects from the parallel thalamus/thalamus:somatosensory cortex (TH/TH:S1:S2) paths, to the posterior insular cortex (pIC), to the medial insular cortex (mIC) and then to DA. The TH and TH:S1:S2 have four US input channels (i.e., up, down, left, and right). A touch event in Figure 1C is one-to-one mapped to a spike in the corresponding channel in TH:S1:S2 where 9-by-8 neurons for each channel match the layout of trackballs. The spikes going through the TH:S1:S2→pIC pathway represent tactile information carried by the fast Aβ-fiber. To model fast spike transmission and acuteness in spatial resolution of this pathway, we topographically connected TH:S1:S2 and pIC as shown in Figure 2C without any delay. In contrast, the spikes going through TH→pIC pathway represent tactile information carried by the slower C-fiber pathway, where a touch event is mapped to a period of tonic spikes. We delayed spike generation in TH by 700 ms to simulate the slower conduction speed of the C-fiber pathway, and fully connected TH to pIC to simulate poor spatial resolution. Since we hypothesize that a piece of tactile information is heterogeneously processed through the parallel paths and then integrated in pIC, the synaptic weights of TH:S1:S2→pIC and TH→pIC were tuned to fulfill the condition that neither TH:S1:S2→pIC nor TH→pIC can dominate the neural activity in pIC. Specifically, the neural activity in pIC was strong enough to drive neural response in mIC and then DA only when there was neural activity in both TH:S1:S2 and TH. As a result, the excitatory force on DA neurons reflects the integration of the neural activity in pIC. To suppress neural activity in pIC after mIC is signaled by pIC, we added feedback connections from mIC to inhibitory neurons in pIC to simulate the effect of shunting inhibition (Silver, 2010). The mIC is linked to the unconditioned response (UR), in which CARL-SJR sings a high tone if mIC has more than 30 spikes within 50 ms.

These complementary excitatory and inhibitory pathways converge on DA neurons. The interaction among STR, INT, and pIC activities controls DA response. The DA responded with a burst (see Section Control CARL-SJR's Tactile Preference through Insular Cortex Model) when the excitatory force was larger, a DA dip (see Section Extinguishing Behaviors after Learning) when the inhibitory force was larger, or spontaneous activity when excitatory and inhibitory forces were balanced. DA is connected to STR and INT through dopaminergic projections. The dopamine values of STR and INT modulate PFC→STR and EXP→INT synapses by DA-STDP and DA-PSF as indicated in Figure 2A.

The synaptic weights, including initial, maximum, and minimum value, were tuned to match the number of pre-synaptic neurons and to prevent run-away neural dynamics. The conductance delays were tuned to maintain stable timing behaviors. The spontaneous firing rates in the cortical regions, ranging from 0.1 to 0.5 Hz (Griffith and Horn, 1966; Koch and Fuster, 1989), were tuned to support baseline neural activities, which are essential for STDP or DA-STDP. The complete network parameters are described in the Supplementary Materials.

### Tactile inputs

**Figure 3:**
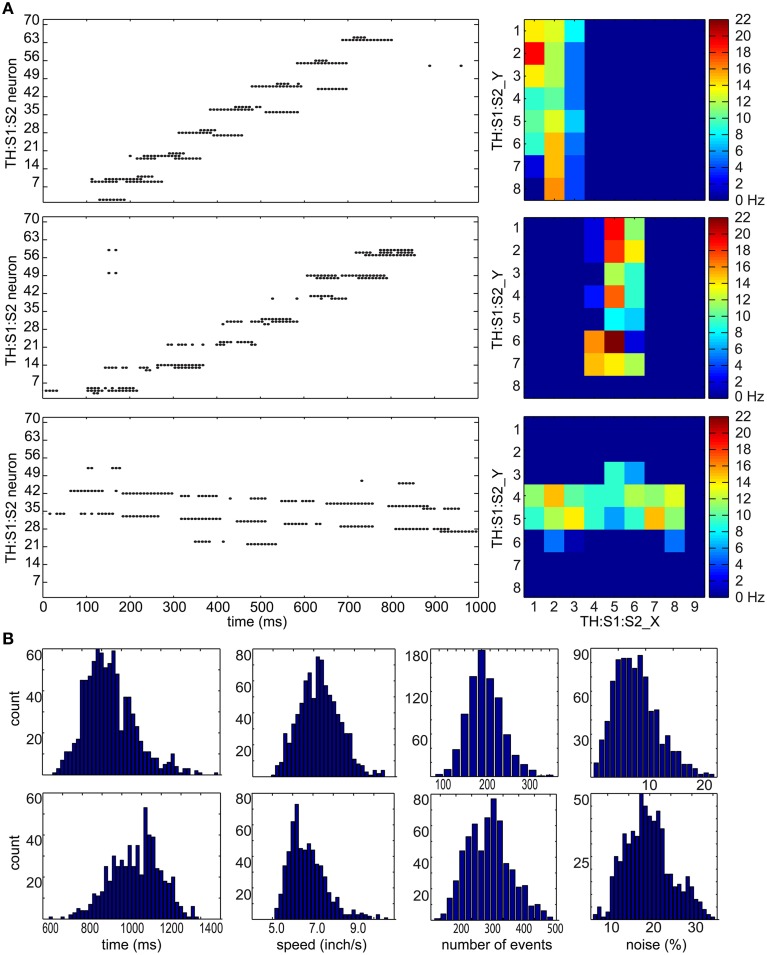
**Tactile stimulus analysis. (A)** Representative spike raster plots and heatmaps of three tactile inputs to the TH:S1:S2 area. On the left are raster plots where the x-axis represents times in milliseconds, and the y-axis represents the neuron number. The top row shows an upward hand sweep on the left side of CARL-SJR's shell, the middle row shows an upward hand sweep in the middle of CARL-SJR's shell, and bottom row shows a rightward hand sweep in the middle of CARL-SJR's shell. The raster plots illustrate how sensory input leads to irregular and noisy spike activity in TH:S1:S2. On the right are heatmaps that show the mean neural activity corresponding to the raster plots. The x and y position in the heat map reflects the somatotopic organization of the TH:S1:S2 neurons. **(B)** The distribution of tactile inputs in contact duration, moving speed, number of events, and noise level (i.e., the amount of unexpected directional moves divided by the expected directional moves). The first row shows analysis based on 800 upward and downward moves while the second row shows analysis based on 600 leftward and rightward moves. The distributions of vertical and horizontal tactile input are quite different because locations of trackballs are asymmetric in vertical and horizontal direction.

We analyzed the properties of tactile inputs (see Figure 3) to gain insight into tactile processing. Figure 3A shows raster plots and heat maps of three tactile inputs: (1) a downward hand sweep on CARL-SJR's left side, (2) a downward hand sweep in the middle, and (3) a rightward hand sweep in the middle. Since a touch event (see Figure 1C) was a one-to-one mapping to spikes in TH:S1:S2 area, the raster plots shows touch events as well. We can see the timing of spikes is irregular and noisy. As for heat maps, they show which trackballs were touched, as well as a somatotopic map in TH:S1:S2 area. Because of the curvature of CARL-SJR's surface, the trackballs were not aligned along the x-axis but mostly aligned along the y-axis (see Figure 1B). Thus, it was harder for users to touch complete rows of trackballs rather than columns of trackball.

Figure 3B shows distributions for touch duration, touch speed, number of touch events, and noise level over 1400 touch movements. The data of upward and downward movements were grouped in the first row while leftward and rightward movements were grouped in the second row because CARL-SJR's asymmetric trackball distribution affected the user's tactile inputs. The (mean, std) of touch durations were (935, 142) and (1,071, 128) ms for vertical and horizontal movement, respectively. The (mean, std) speeds were (7.3, 1.08) and (6.73, 0.93) inch/second for vertical and horizontal movement, respectively. The (mean, std) number of events were (207, 44) and (305, 69) for vertical and horizontal movement, respectively. The (mean, std) noise level were (8.31, 3.97%) and (19.72, 5.53%) for vertical and horizontal movement, respectively, where noise level was defined by the total number of direction events in an unexpected direction divided by the number of direction events in an expected direction. The noise level of horizontal movements is significantly higher due to CARL-SJR's curvature along y-axis.

### Experimental paradigm

**Figure 4:**
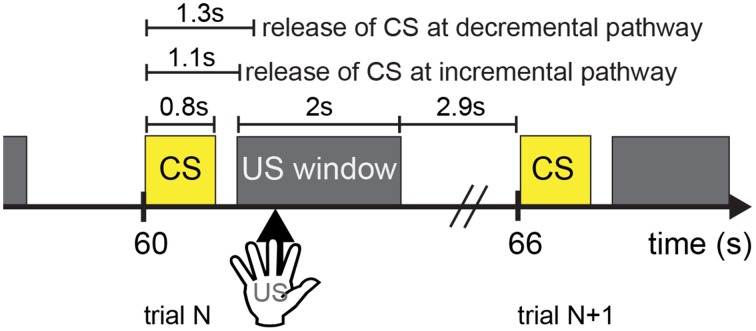
**The timing diagram illustrates the experimental setup**. Each trial lasts 6 s, where a CS signal is followed by a 2-s US window. It is a user's choice to provide a US signal (i.e., tactile input) or not to CARL-SJR. Tactile input outside the US window is unrewarded. After the US window has passed, there is a 2.9-s period before the next trial.

The experiment paradigm is shown in Figure 4. A trial lasted 6 s. CARL-SJR initiated a trial by displaying a color on its surface (see Sections CARL-SJR's Behaviors during Learning Multiple CS-US Pairs and Extinguishing Behaviors after Learning for experimental details). A CS signal corresponding to the displayed color was input to the AG at 1.1 s after trial beginning. Effectively, neural activities were triggered in EXP (i.e., incremental pathway for CS) and PFC (i.e., decremental pathway) at 1.1 and 1.3 s respectively. It was the user's choice to reward CARL-SJR or not. If the user liked the displayed color, he/she could touch CARL-SJR within 2 s US window and the US signal (i.e., tactile input) was delivered to TH and TH:S1:S2, which in turn triggered a dopaminergic reward response (see Figure 2A). Tactile input outside the US window did not activate this reward pathway. After the US window, there was a 2.9 s stabilizing period before the next trial. The experiment continued until learning achieved a desired level (e.g., the probability of CR is higher than UR).

## Results

### Control CARL-SJR's tactile preference through insular cortex model

**Figure 5:**
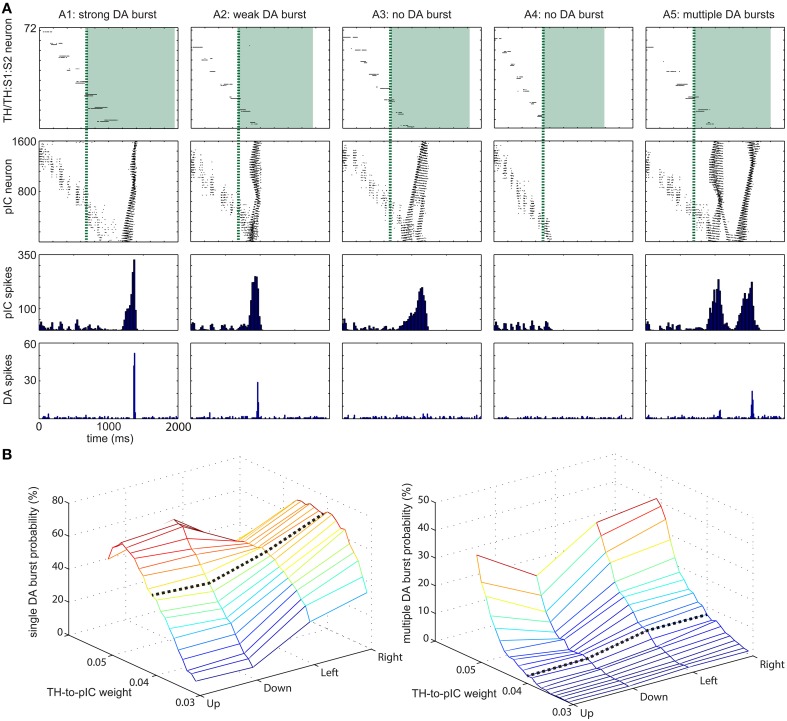
**Range of dopamine (DA) responses to varying input patterns (A) Five representative examples for DA response to tactile inputs**. (A1) strong DA burst. (A2) weak DA burst. (A3) no DA burst due to insufficient instantaneous activity at pIC area. (A4) no DA burst due to no integration of activity at TH and TH:S1:S2 area. (A5) multiple DA bursts. The first row shows raster plots of TH:S1:S2 and TH areas. The spikes of TH area are drawn as green shade regions. The second and third rows show the magnitude of activity at pIC area, which leads to different DA response. The fourth row shows DA response. **(B)** TH→pIC synaptic weights affect DA response. (Left) The probability of a single DA burst based on synaptic weights and the moving direction of the tactile input. (Right) The probability of multiple DA bursts. Setting TH→pIC synaptic weight to 0.04 yields more than 50% of single DA burst while multiple DA burst is lower than 10%.

To characterize the network dynamics in response to sensory input, we ran a set of simulation experiments to explore the SNN's ability to transfer tactile information across different simulated brain regions. The incremental pathway for US was implemented by the model of pIC, which exhibited complicated neural dynamics in response to hand movements. We set TH→pIC synaptic weights to 0.04 for each channel and ran simulations with 400 upward movements as inputs to TH:S1:S2 and TH. Figure 5A illustrates representative neural activities in TH:S1:S2, TH, pIC, and DA. The raster plots in the first row show the spikes directly triggered by touch events in the upward direction. Green boxes indicate the tonic spikes generated by TH in the upward direction, which arrive at pIC 700 ms later because the conductance speed of C-fibers is slower than Aβ-fibers. Before the arrival of tonic spikes, pIC is at the state with low excitability. The spikes from TH:S1:S2 trigger local wavelike neural activity in pIC (see spikes earlier than green dashed lines in the second row). After the arrival of tonic spikes from TH, pIC transitions to a higher excitability state that can trigger global wavelike neural activities in the pIC (see spikes later than green dashed lines). Multiple waves may interact with others and may trigger a DA burst depending on the strength of the instant excitatory force (see histograms in the third row and first three columns for comparison). Note that, TH→pIC synaptic weights are crucial to the excitability of pIC, sustainability of global waves in pIC, and the probability of DA bursts.

The response of pIC was complex and influenced dopaminergic activity. Based on the DA response, we classified neural activities in pIC into five groups: (A1) strong DA burst, (A2) weak DA burst, (A3) no DA burst due to insufficient instant activity in pIC, (A4) no DA burst due to no global activity in pIC. (A5) multiple DA bursts. A gentle hand movement (with moderate speed) is most likely to trigger a DA burst. If the speed of a hand movement is too fast (e.g., the touch duration is less than 700 ms), the neural activity in TH:S1:S2 disappears before tonic spikes from TH tune pIC to excitable state and therefore pIC is unlikely to trigger a DA burst. On the other hand, if the speed is too slow, the excitatory force of TH:S1:S2 is too weak to trigger global waves in pIC even though pIC is tuned to excitable state by tonic spikes from TH.

We adjusted TH→pIC synaptic weights in different channels (i.e., up, down, left, and right) to control the probability of a DA burst linked to CARL-SJR's tactile preferences. To evaluate the probability of a DA burst against different TH→pIC synaptic weights, we ran simulations with 400 upward, 400 downward, 300 leftward, and 300 rightward movements as inputs to TH and TH:S1:S2. For each direction of inputs, we recorded the total number of single DA bursts and derived the burst probability (see Figure 5B). To make CARL-SJR prefer rightward movements, for example, we set the TH→pIC synaptic weights in right channel to 0.04, and the other channels to 0.03. In this case, the probability of a DA burst was greater than 70% for rightward movements but less than 20% for other directions. As a result, CARL-SJR learned faster if the user gives rewards by rightward movements. Figure 5B also shows how the asymmetric trackball distribution affects the number of touch events per hand movement. Note that there are typically more touch events for left and right movements than up and down movements (see the histograms at third column in Figure 3B). This can have an effect on CARL-SJR's tactile preferences. For example, if we set TH→pIC synaptic weights to 0.04 for each channel, horizontal movements yield slightly higher probability of generating a DA burst than vertical movements (see the black dash line in Figure 5B). Under the condition that TH→pIC synaptic weights for each channel are identical, CARL-SJR will prefer horizontal movements. In the experiments described below, the TH→pIC synaptic weights were set to 0.04 for the preferred direction, and 0.03 for the non-preferred direction. Taken together, these simulations showed that the simulated insular cortex could control dopamine bursts, which could lead to the shaping of tactile preferences.

### Wave propagation in PFC

A critical requirement for learning is assigning the credit of a reward to the appropriate stimulus that occurred in the past. Wave propagation has been suggested to be important for computing this timing in classical conditioning tasks (Palmer and Gong, 2014). The idea is that wavelike neural activity in the cortex might encode timing information related to events.

**Figure 6:**
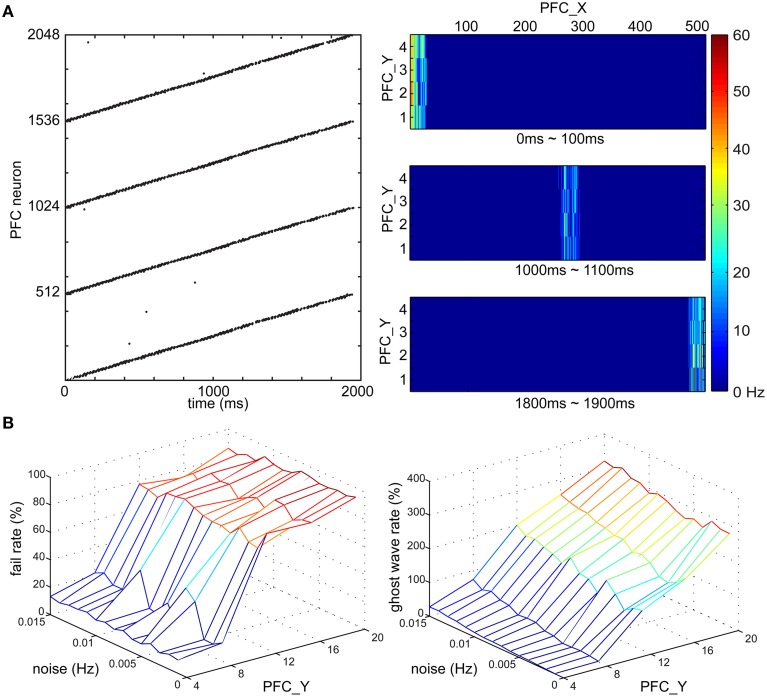
**Time course of PFC wave propagating activity. (A)** Wave propagation in PFC area. (Left) The raster plot shows the time duration of the wave is around 2000 ms. (Right) the wave travels along x-axis of the PFC area and the locations of the wave at three time intervals, 0–100, 1000–1100, and 1800–1900 ms are shown from top to bottom, respectively. **(B)** The fail rate of a wave and the ghost wave rate based on noise (Hz) and the height of PFC area (i.e., the number of PFC neurons along y-axis). The noise level affects the probability of a wave reaching from one end of PFC to another, and on generating a ghost wave. PFC area is more resistant to noise if the height is smaller than 12 neurons.

To investigate if wave propagation was a viable mechanism for the spatiotemporal learning in the present experiments, we incorporated this idea into our simulated PFC and demonstrated traveling waves in a series of simulations (see Figure 6). We modeled PFC as a long rectangular shape and tuned the conductance delay of lateral projections for excitatory neurons to be 15–20 ms and inhibitory neurons to be 1 ms. During a development period, we enabled E/I-STDP and delivered spikes to the most left side of PFC every 4 s. After 2000 s of development, the wave reliably propagated from the left side to the right side of PFC in 2 s as shown by the raster plot in Figure 6A. The heat maps further show the location of a wave at a given time period. After starting the spike activity on the leftmost side of PFC, the wave reached the rightmost side of PFC at 1900 ms. The speed of waves is quite stable because of the lateral conductance delay. As a result, a neuron's location along x-axis encodes time information with high accuracy and reliability.

Noise might stop wave propagation as well as trigger unexpected waves. To address this issue, we tested the robustness of wave propagation under a noisy environment (see Figure 6B). Since we knew when a wave should arrive the most right side of PFC, we defined a fail case to be the absence of neural activity of the most right neurons [i.e., (508, y) ~ (511, y)] at the expected time window (i.e., 1950 ~ 2050 ms). We also defined a ghost wave case to be any occurrence of neural activity outside the expected time window. After the development period, the PFC was tested in 1000 trials where we delivered spikes to the most left neurons, which were connected to AG (see Figure 2B), and then recorded the number of fail cases and ghost wave cases. We derived the fail rate (in percentage) and ghost wave rate against different magnitudes of noise (in Hz) and the height of PFC (in the number of neurons). The fail rate was under 10% if the height of PFC was 4 neurons, and was under 40% if the height of PFC was 8 neurons. However, the fail rate was around 80% when the height of PFC was more than 12 neurons. The magnitude of noise also affected ghost wave rate. The ghost wave rate reached 80% when the height of PFC was 8 neurons and the noise was 0.015 Hz. When the height of PFC was more than 12 PFC neurons, the ghost wave rates were greater than 100%, which meant there were more than one ghost waves in a trial. Both fail rate and ghost wave rate will affect learning efficiency of PFC→STR synapses (see Section PFC-to-STR Synapses Learn to Predict the Timing of US). Therefore, based on these analyses, we set the height of PFC to 4 neurons and set the noise to 0.01 Hz for stable wave propagation.

### PFC-to-STR synapses learn to predict the timing of us

**Figure 7:**
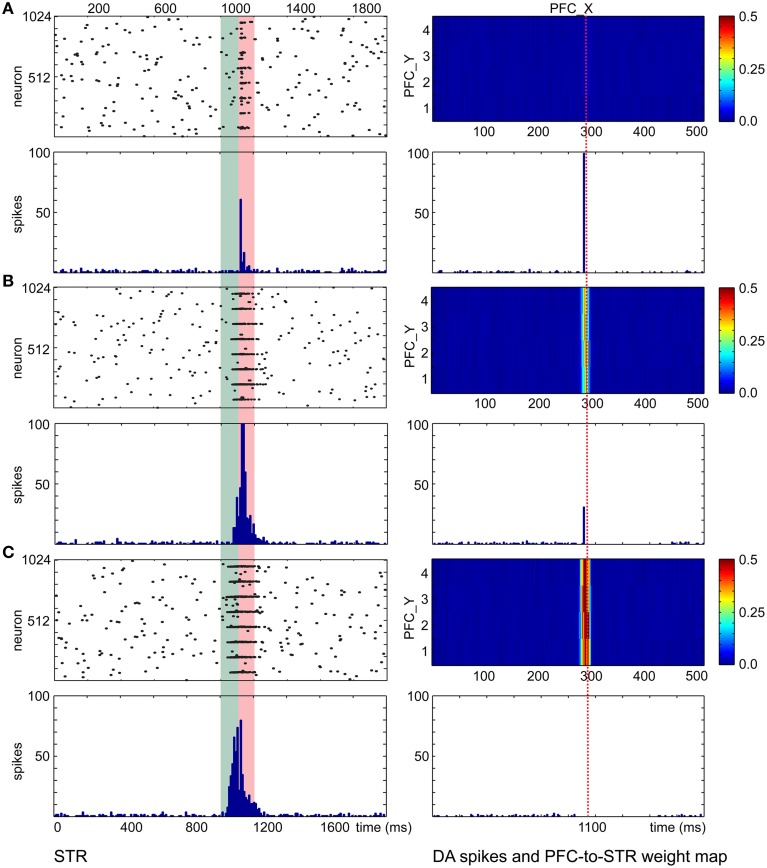
**PFC→STR connection learns the timing of dopaminergic bursts. (A)** Before learning, a DA burst will trigger transient STR activity through dopamine-modulated post-synaptic facilitation. The wave traveling at PFC area matches the STR activity and PFC→STR synaptic weights are reinforced by DA-STDP. The heatmaps on the right of the figure show the PFC→STR weights at different points, where the color represents the summation of the synaptic weights from a PFC neuron to a STR neuron. For instance, the PFC neuron at (290, 2) has 0.5 as the summation of its PFC→STR synaptic weights **(B)** During learning, PFC→STR synaptic weights get stronger and the traveling wave at PFC area can actively trigger spikes at STR area, leading to a weaker DA burst. **(C)** After learning, PFC→STR synaptic weights reach a maximum strength and the inhibitory force from STR area to DA area prevents a DA burst.

With the wave propagation mechanism in place, we still required a means to pair neutral stimuli (e.g., color) with innate value (e.g., a preferred touch). Therefore, we implemented dopamine modulated STDP and wave propagation in the network to associate a CS with a US with precise timing. The main function of the CS input coming from the PFC and STR in the decremental pathway is to predict the timing and strength of the ensuing US and to balance the excitatory and inhibitory forces on the DA neurons. Figure 7 shows simulation results that explain the underlying neural mechanisms. For each trial in the simulation, CS activated the PFC at 0 ms and triggered a propagating wave of activity. A DA burst was activated at 1100 ms to simulate the effect of US. We ran the simulation for 400 trials. The raster plots and histograms of STR activity for trial 1, trial 100, and trial 400 are shown on the left side of Figures 7A–C, respectively. The PFC→STR weights were subject to DA-STDP. The PFC→STR synaptic weights for trial 1, trial 100, and trial 400 are shown on the right side of Figures 7A–C, respectively. In trial 1, a burst in DA increases dopamine concentration in STR through dopaminergic projections around 1100 ms. Because the DA-PSF facilitated STR activity, STR neurons were further activated by pre-synaptic PFC neurons around 1100 ms. Since the CS triggered a wave propagating in PFC, there was always a small portion of PFC firing at any moment. Thus, the set of PFC neurons firing around 1100 ms caused their post-synaptic STR neurons to be potentiated. This neural mechanism led to a phasic neural activity around 1120 ms (due to axonal delay and latency of conductance based synapses) as shown in Figure 7A. The higher dopamine concentration not only facilitated firings of STR neurons but also boosted the PFC→STR synaptic weights through DA-STDP. This process was repeated trial by trial. Over time, the set of PFC→STR synapses with the appropriate timing were strengthened. Comparing the weight maps in Figure 7, a band of strong weights appeared at the PFC neurons encoding 1100 ms (those with x-coordinate from 280 to 300) in Figure 7B and got stronger and earlier (i.e., shift toward the left) in Figure 7C. To summarize, these strong synapses led to phasic neural activity in STR (see STR raster plots and histograms), which in turn suppressed the DA burst at the precise time through the decremental pathway (see DA raster plots).

### CARL-SJR's behaviors during learning multiple CS-US pairs

The previous sections showed through simulation that the SNN could (1) encode tactile patterns, (2) encode timing through propagating waves, and (3) learn associations between neutral and value-laden stimuli. In this section, we show how these mechanisms can be used in real-world human robot interactive learning experiments.

**Figure 8:**
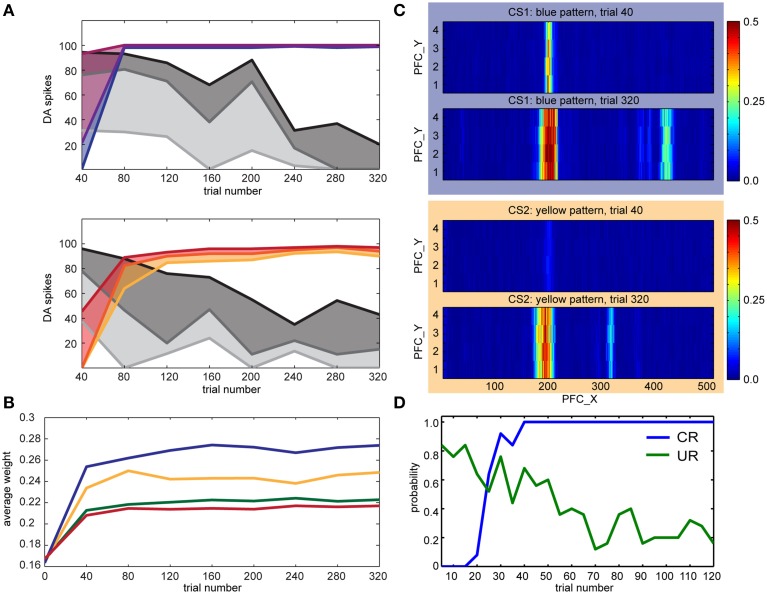
**CARL-SJR learned user preferences for color patterns. (A)** The colored lines show the 75th percentile, median, and 25th percentile of DA response to the CS over 20 trials (totally 40 trials, 20 trials for each color pattern) while the gray lines show DA response to US. **(B)** The average INT→EXP synaptic weights over 20 trials (totally 40 trials, 20 trials for each color during conditioning. The user only reinforced the blue and yellow patterns. **(C)** The PFC→STR weight maps of blue and yellow patterns over trial 40 and trial 320. The interpretation for the heatmap is the same as Figure 7. **(D)** The probability of CR and UR every five trials over five runs. The trends for CR and UR are clear and consistent with the DA spikes triggered by CS and US in **(A)**.

We conducted the conditioning experiments described in Section Experimental Paradigm with different pairings of color and touch. In the first experiment (see Figure 8), we set TH→pIC synaptic weights to 0.04 and CARL-SJR's display alternated between blue, and yellow. The user rubbed CARL-SJR in the downward direction whenever he/she saw the yellow pattern, and in the rightward direction when he/she saw the blue pattern. There were 160 trials for each color pattern, totally 320 trials. We recorded the DA responses for US and CS every trial and sampled the PFC→STR synaptic weights every 40 trials. The colored lines in Figure 8A show the 75th percentile, median, and 25th percentile of DA response to the CS over 20 trials (totally 40 trials, 20 trials for each color pattern) while the gray lines show DA response to US. The trends for CS and US are clear; DA response shifted from US to CS for both color patterns as has been observed in empirical studies (Ljungberg et al., 1992; Schultz, 1998; Pan et al., 2005). Figure 8B shows the average EXP→INT synaptic weights for each CS (i.e., red, green, blue, and yellow) during conditioning. Since the user only reinforced the blue and yellow patterns, the average synaptic weights directly reflect the user's preferential conditioning as shown by the higher values of blue and yellow EXP→INT weights. The PFC→STR weight maps of trial 40 and trial 320 are shown in Figure 8C. These weight maps, which were driven by the user conditioning CARL-SJR, exhibit a strong group of weights associated with the CS with several bands of weights to a lesser degree. The width of a band indicates the imprecise timing of the US and the strength of a band indicates the probability of a US occurrence at the corresponding timing. The DA response to horizontal movements (i.e., US) decreased faster than vertical movements due to the stronger weights for the blue pattern. The weight maps generated by real time tactile inputs also demonstrated our approach can capture the timing and strength of US over a wide range and in a noisy, real-world environment. An interesting behavior is that CARL-SJR slightly prefers touches in the horizontal, rightward direction. In Section Control CARL-SJR's Tactile Preference through Insular Cortex Model, we showed that CARL-SJR by default prefer horizontal movements if we set TH→pIC synaptic weights to 0.04 for all channels. Because of this asymmetry, CARL-SJR shifted DA response to blue pattern faster than yellow pattern (see the higher blue median value in Figure 8A and the higher blue line in Figure 8B) by the default preference. The result here is also consistent with Section Control CARL-SJR's Tactile Preference through Insular Cortex Model.

In a second set of experiments, we focused on the robot's behavior in the form of conditioned and unconditioned responses. For the conditioned response, which was based on INT activity, CARL-SJR rotated its body. For the unconditioned response, which was based on mIC activity, CARL-SJR emitted a high tone. The US was a downward movement, and the CS was the yellow pattern. We collected data for five runs and each run contained 120 trials. Each data point in Figure 8D was calculated as the probability of CR and UR every five trials over five runs. The trends for CR and UR are clear and consistent with the DA spikes triggered by CS and US (see Figure 8A). CARL-SJR learned to exhibit the CR with high probability after 40 trials and also suppressed the UR after around 70 trials. Taken together, these human-robot interaction experiments show that the proposed mechanisms can support learning in a real-world, noisy environment.

### Extinguishing behaviors after learning

**Figure 9:**
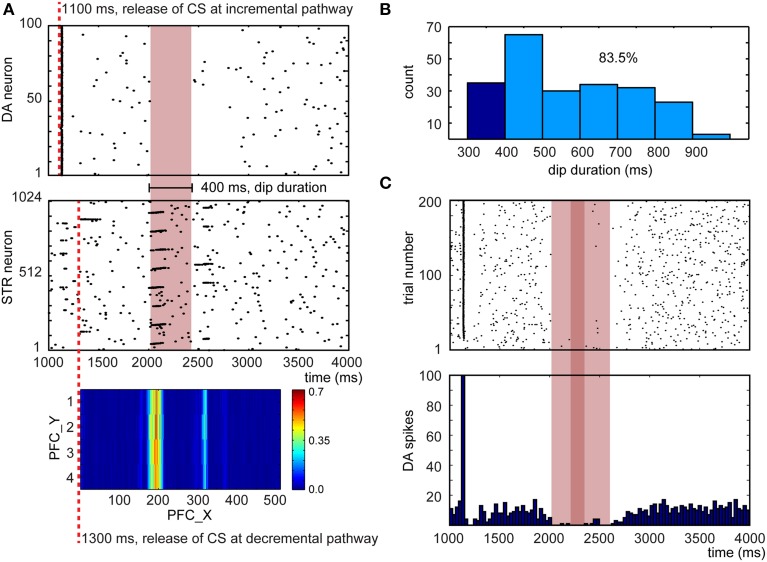
**Network response after learning when the US is omitted. (A)** A representative example for a dip in DA response due to the absence of expected rewards. The heatmap at the bottom shows the PFC→STR weights, where the color represents the summation of the synaptic weights from a PFC neuron to a STR neuron. **(B)** Distribution of dip durations in 200 trials. The threshold for triggering a low tone is set to 400 ms. 83.5% trials trigger a low tone. **(C)** The raster plot and histogram of 5 randomly selected DA neurons over 200 trials when the US is omitted.

The ability to unlearn prior associations is critical for flexible behavior. In conditioning paradigms, omitting the US after learning can lead to extinction of the conditioned response. After conditioning, we repeated the experiment described in Section CARL-SJR's Behaviors during Learning Multiple CS-US Pairs without presenting the US in an additional 200 trials. The DA response and CARL-SJR's behavior were recorded as well. Figure 9A shows a representative example for a dip of DA response due to the absence of expected rewards (Ljungberg et al., 1991). The raster plot of DA spikes shows the phasic neural activity around 1100 ms, which was triggered by CS via the incremental pathway. At 1300 ms, the CS arrived at PFC and triggered wave propagation. Based on the weight map developed during learning process, STR neurons exhibited a phasic neural activity around 2000 ms. Since no excitatory force was present around 2000 ms (due to absence of US), the inhibitory force from STR suppressed DA neurons and created a 400 ms interval without DA spikes. In Figure 9B, we show the distribution of dip durations in 200 trials. In these experiments, if there was dip in DA activity longer than 400 ms, CARL-SJR emitted a low tone signaling the omission of an expected reward. During this experiment we observed CARL-SJR sang this unhappy low tone in 167 out of 200 trials, which is 83.5%.

To emulate the *in vivo* recordings from Ljungberg's experiments (Ljungberg et al., 1991), we randomly selected 5 out of 100 DA neurons over 200 trials. This emulated recording from a small sample of the available pool of dopaminergic neurons. From these recordings, we composed a raster plot and a histogram in Figure 9C. The dip duration is roughly 500 ms (see light red bar in Figure 9C) and there is no DA activity for around 100 ms (see deep red bar in Figure 9C). These dip durations closely matches those reported in Ljungberg's study.

**Figure 10:**
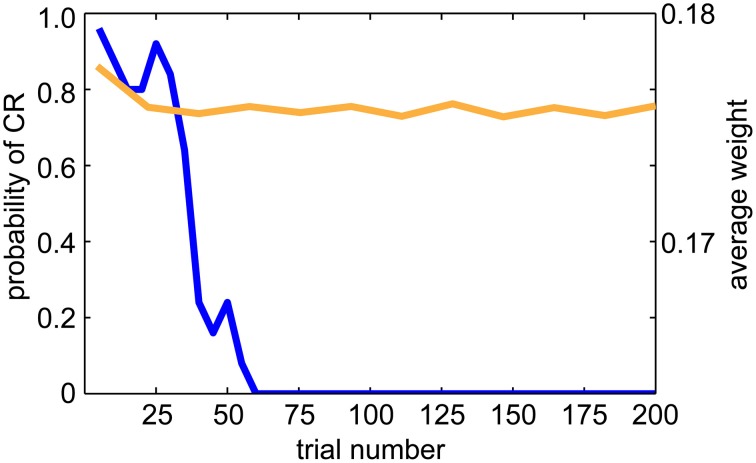
**Extinction behavior when the US is omitted**. The blue line shows the average CR probability every 5 trials over 5 runs during the extinction trials. The probability of CR decayed to 0.0 after 50 trials, which is consistent with animal studies. The orange line shows the average EXP→INT synaptic weights for the reinforced color pattern. Note that the weights decay to a baseline level during extinction.

The DA dips, shown in Figure 9, have been suggested to promote extinction when the reward associated with the US is absent. Therefore, we tested extinction behavior in a classical conditioning paradigm (see Figure 10). After conditioning, we repeated the experiment without presenting the US. We then recorded the CR and UR for 5 runs and each run had 200 trials. We calculated the probability of the CR every 5 trials over 5 runs. As is indicative of extinction of a behavior, the probability of CR decayed to zero after 50–60 trials (see blue line in Figure 10). We also plotted the average EXP→INT synaptic weights for the yellow pattern (see orange line in Figure 10). The weights decayed and exhibited fluctuation around a steady level. The decay was due to dips of dopamine (see Figure 9B) and noisy spontaneous firing activities in EXP and INT.

## Discussion

We introduced a tactile neurorobot, called CARL-SJR, which was capable of sensing noisy, real-world tactile inputs in a highly uncertain environment and taking tactile inputs with minimal pre-processing to convert touch events into spiking activity (see Section Control CARL-SJR's Tactile Preference through Insular Cortex Model). A detailed spiking neural network (SNN) model of somatosensory cortex and the insular cortex, which is known to be important for hedonic touch, drove CARL-SJR's behavior. Learning in the model was driven by a dual pathway model of dopaminergic learning and the emergence of traveling waves of neural activity that governed the release of dopamine and the timing of CS and US (see Sections Wave Propagation in PFC and PFC-to-STR Synapses Learn to Predict the Timing of US). CARL-SJR demonstrated the ability to associate multiple CS's (i.e., color patterns displayed on its shell) with US's (i.e., user hand sweeps across its shell). For example, in Section CARL-SJR's Behaviors during Learning Multiple CS-US Pairs, we showed that the model supported associations between blue-right and yellow-down during a training session. Moreover, the model was able to learn despite trial-by-trial variations in CS-US intervals due to the uncertainties of user inputs.

The human-robot interaction studies with CARL-SJR exhibits the paradigm of mutual reinforced learning. Specifically, the robot can learn the user's preferences through conditioning tasks while the user can learn the robot's tactile preference through the robot's responses. CARL-SJR's style of human-robot interaction may be applications in socially assistive robotics (Scassellati et al., 2012) or socially affective robots (Breazeal, 2009). Compared to other social robots, CARL-SJR is somewhat unique in that it focuses on tactile rather than visual interaction.

CARL-SJR was designed to encourage interaction through touch. Tactile sensing is an active area of robotics research that takes inspiration from biology and neuroscience. For example, inspired by rodents and other mammals with vibrissae, whiskered robots have been developed to sense the borders and shape of objects (N'Guyen et al., 2010; Pearson et al., 2011; Evans et al., 2012; Schroeder and Hartmann, 2012). Fingers and hands have been developed for humanoid robots to enable grasping and detecting surfaces (Bologna et al., 2011, 2013; Spigler et al., 2012; Li et al., 2013). Most of these robots are constructed from custom-made materials and sensing circuits for touch (Sewards and Sewards, 2002; Cannata et al., 2008; Maheshwari and Saraf, 2008; Dahiya et al., 2010). Creating an artificial tactile system is difficult for many reasons. For example, the sensors must cover a large range and be compliant with the surfaces with which they interact. Moreover, the spatiotemporal nature of tactile stimuli, as well as the noisy sensors and environments in which they operate, make the perception of touch a complex problem. To provide a large surface that could handle a wide range of user inputs, we utilized a matrix of trackballs, which are found in many cellphones, across CARL-SJR's curved shell. The size, shape, and resolution was a good fit for the types of social interactions for which CARL-SJR was designed (Bucci et al., 2014).

Driven by the dual-pathway model, the acquisition and extinction behaviors of CARL-SJR are consistent with animal behavioral studies (Rescorla, 1988). Further, the neural activities in our model are consistent with *in vivo* neural recordings as well. First, the robot has built-in tactile preferences. This matches the animals' behaviors wherein they prefer social touches in particular areas and directions. The neural mechanism for integrating tactile information in pIC area could serve as an explanation to animals' innate tactile preferences. Second, assuming the user knows the tactile preferences in advance or learns them, the user can try to reward the robot by touching the robot in its preferred ways. In the present experiments, the unconditioned response (UR) was mapped to mIC activity resulting in a high tone signaled by the robot. The high tone was used as feedback for the user to know that CARL-SJR enjoyed this touch. Third, the robot can learn the association between a color and a gentle touch. In the present paper, a color displaying on the robot's surface, which was spontaneously generated, was considered the conditioned stimulus (CS), and a gentle touch in an innate preferred direction (i.e., unexpected reward) served as the unconditioned stimulus (US). After learning, the robot associated the reinforced color, which was facilitated by DA, with the future reward, which is a preferred touch. Moreover, the DA response shifted from US to CS, as has been observed experimentally (Ljungberg et al., 1992; Schultz, 1998; Pan et al., 2005). CARL-SJR expressed its conditioned response (CR) when INT activity was high and signaled the CR with a spinning motion and bright colors displayed on its surface. Fourth, the robot was depressed if a gentle touch was expected, but not given. This occurred as a dip in DA activity when the expected US was withheld. We mapped the duration of a dip to a low tone, which sounded unhappy. This behavior was triggered by a dip of dopamine concentration level (Ljungberg et al., 1991).

### Comparison with previous computational models

To associate temporally separated events (i.e., CS and US), many computational models assumed the firing activities of neurons responding to an earlier event slowly decay and the sustained firing activities are associated neurons with the ensuing US event through STDP (Gluck and Thompson, 1987; Drew and Abbott, 2006). Similarly, the slowly decaying eligibility trace (Houk et al., 1995) has been applied to associate temporally separated events in dopamine modulated STDP (Izhikevich, 2007). The idea of slowly decaying eligibility trace was also successfully applied to rate-based neurons where Soltoggio and Steil used rare neural correlations to calculate the eligibility trace (Soltoggio and Steil, 2013). This approach was further validated on iCub for classical and operant conditioning tasks (Soltoggio et al., 2013). Chorley and Seth later integrated the DA-STDP mechanism into the dual-pathway model (Chorley and Seth, 2011). Their model successfully accounted for a wide range of reward-related DA responses. A different approach to conditioning paradigms is to incorporate temporally separated events as propagating spiking waves and associate these events through the spatiotemporal interaction of these waves (Palmer and Gong, 2014).

Using wave propagation to solve the temporal credit assignment problem has some interesting features that address limitations in other neurobiologically plausible reinforcement learning rules. The early dopamine model of reinforcement learning was very similar to temporal difference learning (Montague et al., 1996; Schultz et al., 1997). From empirical data and computational modeling, dopamine appeared to track the reward prediction error. However, in the model, the dopamine signal moved backward in time in successive trials until it corresponded to the stimulus that was predictive of reward. This movement of a dopamine signal over time has not been observed empirically. Others have proposed an eligibility trace as a means to solve the credit assignment problem (Izhikevich, 2007; Soltoggio and Steil, 2013). However, since the amplitude of the trace from the time of the CS to the time of the US can be quite small, it requires many trials to make a strong association. Moreover, there is little empirical support for a biological process to support this type of learning that lasts over many seconds. In contrast, wave propagation has empirical support and does not have the limitations described above (Rubino et al., 2006; Benucci et al., 2007; Ferezou et al., 2007; Han et al., 2008; Wu et al., 2008; Lubenov and Siapas, 2009; Sato et al., 2012). These waves have the appropriate timescale, are robust to variations in timing, and can send a strong enough signal to support associative learning in a plausible number of trials.

Another interesting approach to solving the credit assignment was proposed by Khamassi et al. (2011), where dopamine neurons produced a reward prediction error signal in response to any salient event and affected synaptic plasticity when it co-occurred with a motor efference property. This would also address the limitations described above. However, in our present experiments we do not have a motor efference copy. The CS is a color display on the robot's shell and does not produce a motor action. The motor command is actually from the subject interacting with the robot. Still, this may be an interesting approach to implement in future models.

CARL-SJR's SNN model, which integrated the slow C-fiber and fast Aβ-fiber tactile pathways to the pIC, Chorley and Seth's dual-pathway model, and Palmer's spiking wave propagation, demonstrated associative learning in the real-world with a robot receiving noisy user input. Several prerequisites or neural behaviors in Chorley's and Palmer's work limit their real-world applications. Palmer's model successfully shifts the neural response from US to CS. However, the US could trigger both a CR and a UR at the same time if the CS is not presented before US. PFC activity in Chorley and Seth's model was implemented with a pre-generated polychronous group (Izhikevich, 2006). The appearance of consistent polychronous groups in a noisy environment is difficult. For example, the criteria for re-occurrence of a polychronous group was set to a low threshold (i.e., 25% neurons of a group) in Szatmary and Izhikevich (2010). In this case, only a small portion of neurons showed time-locked spike patterns. In contrast, when the criteria for re-occurrence was set to a high threshold (e.g., 100% neurons of a group) as in Bucci et al. (2014), the polychronous groups were very small, rarely occurred, and lasted for less than 40 ms. In this prior neurorobot study, these polychronous groups did not match the PFC activity in Chorley's model where time-locked cortical spike patterns sustained for 1 s. Both Chorley's and our model exhibit an interesting neural mechanism in which the STR response was a little bit later than the DA response in early trials (see Figure 7A) and then shifted backward to match the timing of the DA response in late trials (see Figure 7C). Whereas Chorley and Seth did not discuss how the PFC→STR synaptic weights may affect the learning progress, we showed the PFC→STR weight maps were indicative of the US timing and this may support temporal associations.

Our present PFC model incorporated the idea of spiking wave propagation in Palmer's model, which made pre-generated spikes or pre-processing of the CS unnecessary. The location of a neuron in PFC and the topographical projections to STR encoded the time relative to the CS in the decremental pathway. The PFC→STR weight map clearly reflected the learning status (see Figure 7). The firing rate of our PFC model is consistent with experimental studies: <0.5 Hz in the resting state (Koch and Fuster, 1989) and 5–40 Hz when behaving (Funahashi et al., 1989). Further, our model has been validated to handle the uncertainty of CS-US interval within 2 s while Chorley reported the valid US window to be (500 ± 100 ms). We also used inhibitory fast spiking neurons to simulate striatal medium spiny neurons (Humphries et al., 2009). The characteristic short spiking burst of a FS neuron facilitated learning in the decremental pathway. The FS neuron transitioned to an excitable state due to DA-PSF, and activity from a pre-synaptic RS neuron in PFC triggered a spike train in the post-synaptic FS STR neuron. This resulted in multiple increases in the synaptic weights through LTP. This scenario was dependent on increased dopamine activity.

The model guiding CARL-SJR's behavior was also unique in that it implemented the separate and parallel pathways for transmitting touch information to the cortex, in which fine touch is well represented in the somatosensory cortex, and hedonic caressing appears to represented in the insular cortex (Sewards and Sewards, 2002; Olausson et al., 2010; Morrison et al., 2011a). The response of the fast Aβ-fiber tactile pathway is reflected in the fine resolution somatotopic response shown in Figure 3A. Less is known about insular neuronal activity in response to touch. However, it has been suggested that the insular cortex can make predictions of internal states (Singer et al., 2009; Seth, 2013) and the neural architecture driving CARL-SJR's pleasure seeking behavior supports these claims (see Figure 2).

### CARL-SJR's limitations

CARL-SJR has several limitations. We developed a PFC wave with relatively low internal noise (i.e., spontaneous neural activity). The maximum noise in Figure 6B is 0.015 Hz, which is 10 times smaller than the spontaneous firing activity (i.e., white noise) in resting PFC suggested by the experimental study of Koch and Fuster (1989). The low spontaneous firing activity extremely slows down the extinction process in the conditioning task (see Section Extinguishing Behaviors after Learning) because the probability to decorrelate coupled neurons in PFC and STR is too low. We left this issue to a future improvement on developing wave propagation under a typical noise level.

The peak dopamine value in our model is 20 μM, which is much higher than 3 μM reported in Izhikevich's and Chorley's models. The range of dopamine value was tuned to result in adequate learning rates in the neurorobot experiments. We could lower the dopamine value and keep the observable learning speed if some compensatory neural mechanisms were implemented. For example, a replay mechanism for the CS and US pairings when CARL-SJR “sleeps” (Buzsaki, 1998). However, to incorporate a true replay mechanism would require substantial efforts in building a hippocampus model for offline learning (Khamassi and Humphries, 2012). A simple approach, which would be effective in the present architecture, would be to simulate replay by injecting the CS and US into the model when CARL-SJR is not actively behaving.

The neural response of mechanoreceptors with C-fiber is tuned for gentle speeds (Morrison et al., 2011a). We did not capture this characteristic because the trackballs, as they are currently designed, cannot detect speed locally (i.e., the number of touch events is not proportional to the rolling speed of a trackball). We could calculate the speed of a movement across multiple trackballs. However, this approach requires substantial pre-processing and therefore, contradicts our design choice.

## Conclusions

CARL-SJR is a neurorobot whose behavior is guided by a SNN model of tactile pathways in the cortex, and demonstrates user defined entraining of a robot through touch. By incorporating dopamine modulated learning with traveling waves of neural activity, we have shown a biologically plausible method of instrumental conditioning. Our SNN model can be easily extended for robotic applications in reinforcement learning paradigms. Future directions include dopaminergic projections to the frontal cortex and implementation of the serotoninergic (5-HT) system (Krichmar, 2013). DA is linked to rewards and curiosity-seeking behavior and 5-HT may be linked to risk aversion and withdrawn behavior (Tops et al., 2009; Boureau and Dayan, 2011; Siegel and Crockett, 2013). It has also been suggested that 5-HT plays a role in waiting for a delayed reward (Miyazaki et al., 2012). By combining the DA and 5-HT systems, we could make CARL-SJR not only learn through rewards but also cost. Moreover, following the notion of 5-HT being related temporal discounting, we could use 5-HT levels to modulate CARL-SJR's impulsiveness. These additions would make CARL-SJR's behavior more interesting and such a system could have applications in the fields of socially assistive and socially affective robotics.

### Conflict of interest statement

The authors declare that the research was conducted in the absence of any commercial or financial relationships that could be construed as a potential conflict of interest.
