# Supplementary material for: Learning touch preferences with a tactile robot using dopamine modulated STDP in a model of insular cortex
Source: Front Neurorobot. 2015 Jul 22;9:6. doi: 10.3389/fnbot.2015.00006 (PMC4510776; doi:10.3389/fnbot.2015.00006)
Supplement: Supplementary file 1 [file DataSheet1.PDF]

Supplementary Material

Summary of Network Parameters

Table S1. Summary of the parameters of neural group in Section 2.2.3

Group Label	Group Size	Neuron Type	Location	Density	Noise
ag{R,G,B,Y}	4 x 4	Spike Generator	(0,0)	(100,100)	N/A
pfcExc{R,G,B,Y}	512 x 4	Excitatory RS	(0,0)	(100,100)	0.001
pfcInh{R,G,B,Y}	128 x 2	Inhibitory FS	(0,0)	(200,200)	0.001
Str	128 x 8	Inhibitory FS	(0,0)	(400,50)	0.1
Exp	16 x 16	Excitatory RS	N/A	N/A	0.5
Int	16 x 16	Excitatory RS	N/A	N/A	0.5
thU	9 x 8	Tonic Spike Generator	(800,800)	(800,800)	N/A
thD	9 x 8	Tonic Spike Generator	(1600,800)	(800,800)	N/A
thL	9 x 8	Tonic Spike Generator	(800,1600)	(800,800)	N/A
thR	9 x 8	Tonic Spike Generator	(1600,1600)	(800,800)	N/A
thS1S2U	9 x 8	Spike Generator	(800,800)	(800,800)	N/A
thS1S2D	9 x 8	Spike Generator	(800,1600)	(800,800)	N/A
thS1S2L	9 x 8	Spike Generator	(1600,800)	(800,800)	N/A
thS1S2R	9 x 8	Spike Generator	(1600,1600)	(800,800)	N/A
picExc	40 x 40	Excitatory RS	(0,0)	(200,200)	N/A
picInh	20 x 20	Inhibitory FS	(0,0)	(400,400)	N/A
Da	10 x 10	Dopaminergic RS	N/A	N/A	0.5

RS neuron a = 0.02, b = 0.2, c = -65.0, and d = 8.0

FS neuron a = 0.1, b = 0.2, c = -65.0, and d = 2.0.

Decay time constant τ_i = 5, 100, 6, and 150 ms where i = {AMPA, NMDA, $GABA_A$, $GABA_B$}

Decay time constant τ_d = 50 ms

Decay time constant τ_c = 1000 ms

Table S2. Summary of the parameters of connections in Section 2.2.3

Source Group	Destination Group	Synapse Type	Weight Range	Connect Type	Radius / Probability	Delay Range
ag{R,G,B,Y}	pfcExc{R,G,B,Y}	E-Fixed	0.06	Radius	400	(10,15)
ag{R,G,B,Y}	pfcInh{R,G,B,Y}	E-Fixed	0.02	Radius	400	(15,20)
pfcExc{R,G,B,Y}	pfcExc{R,G,B,Y}	E-STDP	(0,0.022,0.06)	Radius	400	(15,20)
pfcExc{R,G,B,Y}	pfcInh{R,G,B,Y}	E-STDP	(0,0.022,0.06)	Radius	400	(15,20)
pfcInh{R,G,B,Y}	pfcExc{R,G,B,Y}	I-STDP	(0,-0.01,-0.03)	Radius	450	(1,1)
pfcExc{R,G,B,Y}	str	DA-STDP	(0,0.001,0.03)	Radius	400	(10,10)
str	da	I-Fixed	0.005	Random	0.4	(1,10)
ag{R,G,B,Y}	exp	E-Fixed	0.03	Random	0.2	(1,10)
exp	int	DA-STDP	(0,0.001,0.008)	Random	0.2	(1,10)
int	da	E-Fixed	0.006	Random	0.4	(1,10)
thS1S2{U,D,L,R}	picExc	E-Fixed	0.1	Radius	400	(1,20)
thS1S2{U,D,L,R}	picInh	E-Fixed	0.05	Radius	600	(1,20)
th{U,D,L,R}	picExc	E-Fixed	0.0004	Full	1.0	(1,1)
picExc	picExc	E-Fixed	0.028	Radius	400	(1,20)
picExc	picInh	E-Fixed	0.02	Radius	400	(1,20)
picInh	picExc	I-Fixed	-0.008	Radius	600	(1,1)

picExc	da	E-Fixed	0.004	Full	1.0	(10,10)
da	picInb	E-Fixed	0.004	Full	1.0	(10,10)
da	str	DAergic	0	Random	0.04	(1,10)
da	int	DAergic	0	Random	0.2	(1,10)
